# Supplementary material for: Microfluidic chips provide visual access to in situ soil ecology
Source: Commun Biol. 2021 Jul 20;4:889. doi: 10.1038/s42003-021-02379-5 (PMC8292388; doi:10.1038/s42003-021-02379-5)
Supplement: Supplementary file 2 — Supplementary Information [file 42003_2021_2379_MOESM2_ESM.pdf]

Supplementary Information

Microfluidic chips provide visual access to *in situ* soil ecology

Paola Micaela Mafla-Endara[1,2], Carlos Arellano-Caicedo[1], Kristin Aleklett[1,3], Milda Pucetaite[1], Pelle Ohlsson[4], and Edith C. Hammer[1,2*]

[1]*Department of Biology, Lund University, Lund, SWEDEN*

[2]*Centre for Environmental and Climate Science, CEC, Lund University, Lund, SWEDEN*

[3]*Department of Plant Protection Biology, Swedish University of Agricultural Sciences, Alnarp, SWEDEN*

[4]*Department of Biomedical Engineering, Lund University, Lund, SWEDEN*

**corresponding author: Edith Hammer, Microbial Ecology, Department of Biology, Lund University, Ecology Building, Sölvegatan 37, 223 62 Lund, Sweden, +46 (0)732 44 1968,*

edith.hammer@biol.lu.se

Supplementary Figures

a

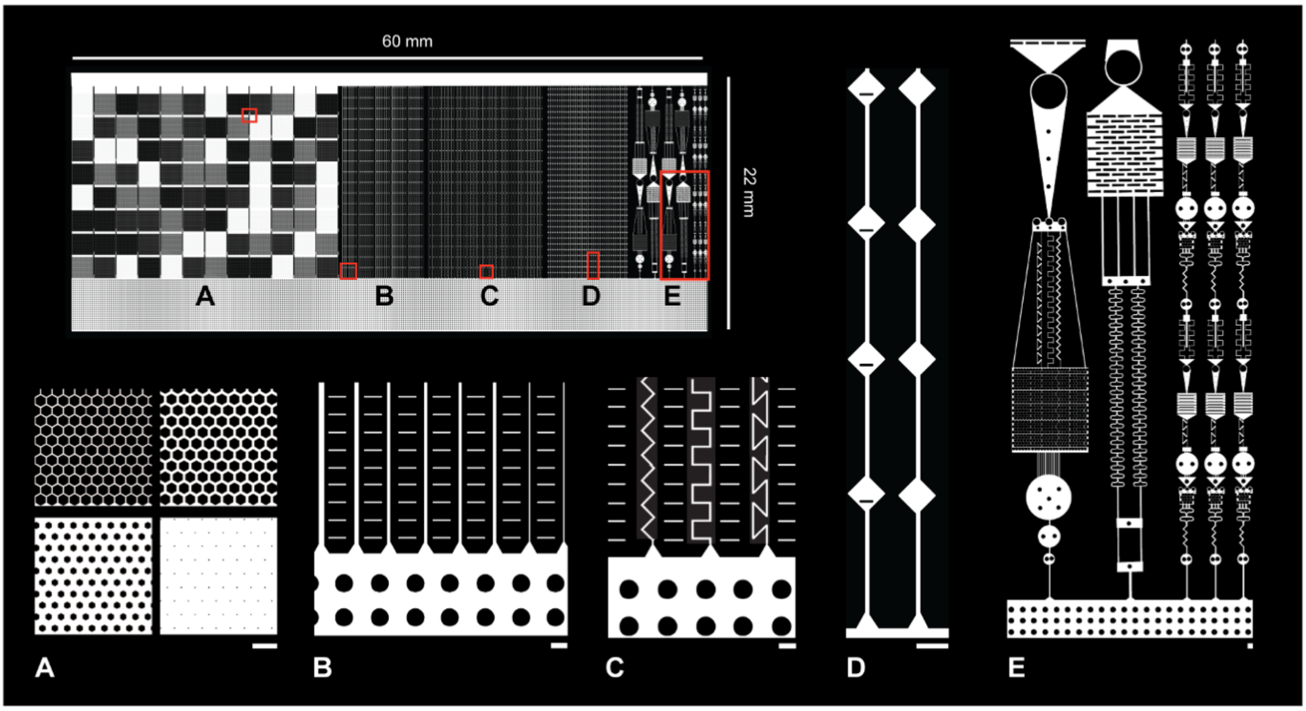

b

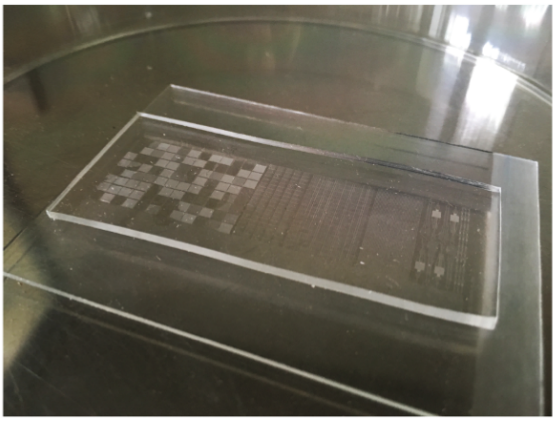

c

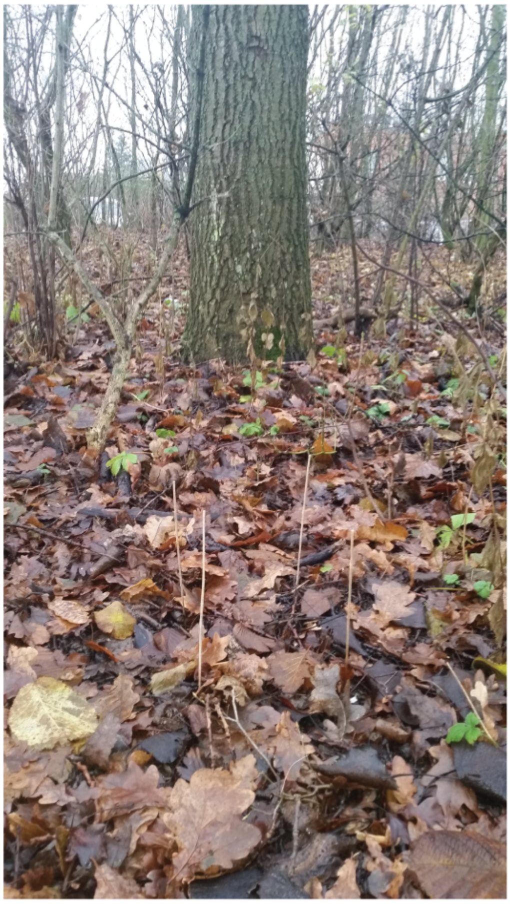

d

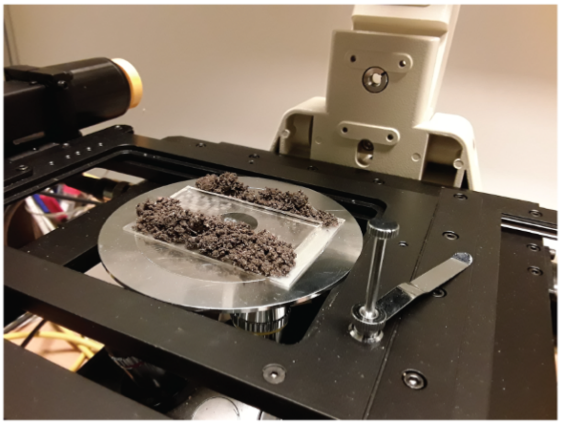

Supplementary Figure 1. The soil chip and its use in soil. a Overview over the design of the chip's internal structures, which contains five particular experimental sections, A-E as depicted in the top, and with detailed, zoomed-in illustrations for each section below and to the right. The red squares indicate the position of the high-magnification illustrations in the chip overview for each respective experimental section. Section A represents blocks of hexagonal pillars with different diameters thus creating different porosities (n=24). Section B consists of straight channels of different widths (20, 15, 10, 8, 6, 4 µm) with five replicates of each type. Section C corresponds to a set of 10 µm wide angled channels with three different types of turning angle/arrangement (zigzag, square and z-shaped, n=12). The segments in between the angles in zigzag and square design are 100 µm; the long segments of the z-shaped channels are 200 µm long. The total length of the zigzag channels is 27000 µm, the other channel types were analyzed to the same length only. Section D consist of repeated patterns of 10 µm width straight channels interspersed by widenings of a diameter of 140 µm. Section E includes two different obstacle courses containing complex structures in irregular arrangement. Scale bars = 100 µm (Section A, B, C, D, E). **b** Photograph of a newly produced chip. **c** Site where a chip was buried into the soil at 10 cm depth and remained for two months. **d** Microscopy analysis of the chip with adjacent soil after unearthing.

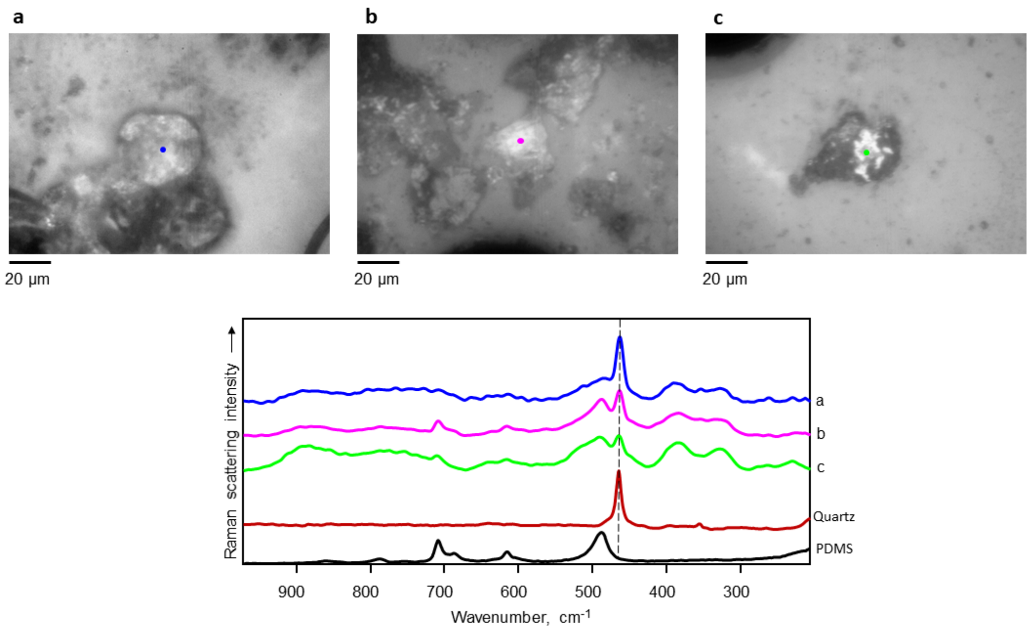

Supplementary Figure 2. Raman scattering microscopic analysis of mineral particles inside the chip. Examples of measured minerals (**a-c**), and their corresponding spectra over the reference spectra for quartz and PDMS.

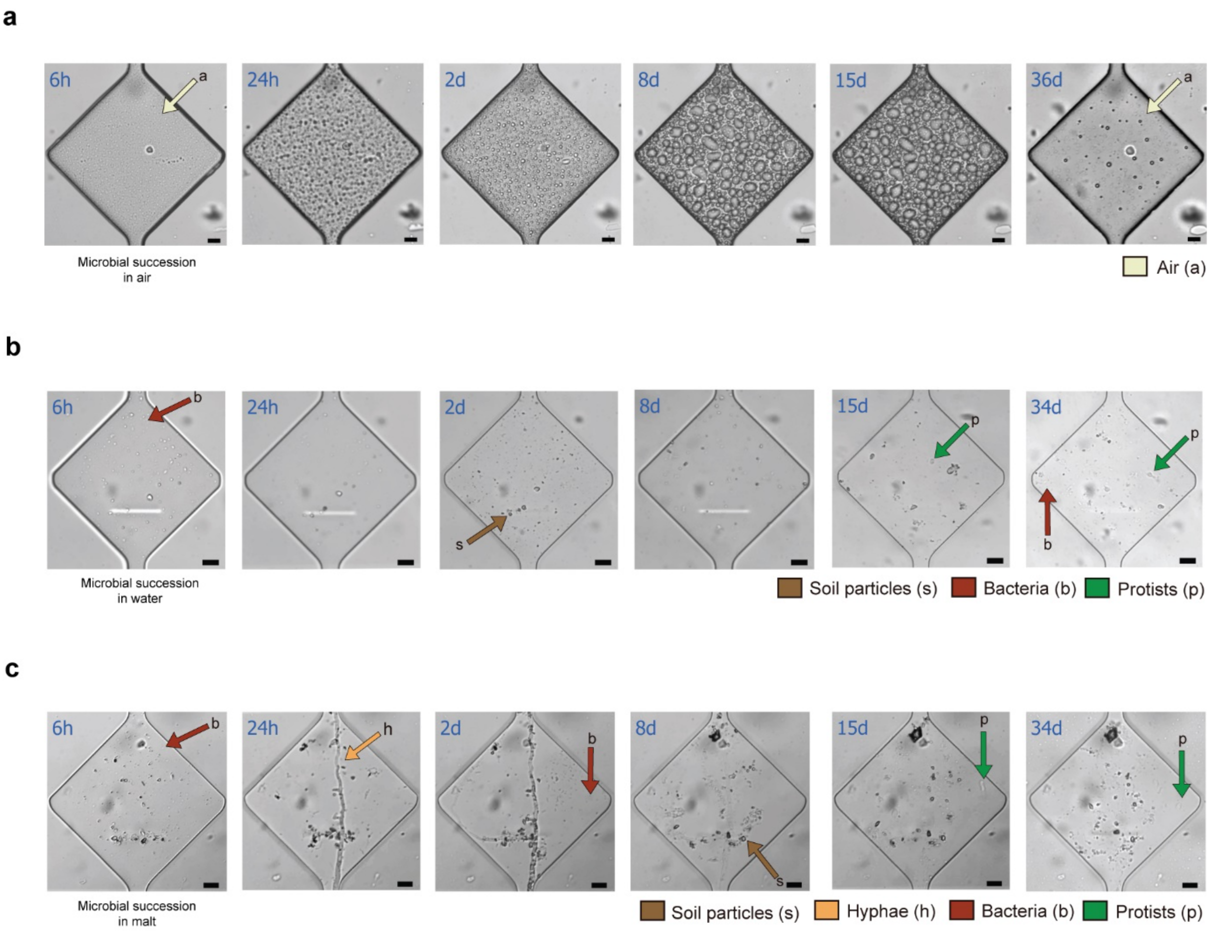

Supplementary Figure 3. Microbial succession in the soil chips incubated with soil in the laboratory (Expt. 2). Image sequences of the time-resolved changes in microbial colonization of a diamond-shaped widening along the channels used to calculate the presence of major soil microbial groups and water films in chips filled with air (**a**), water (**b**) and malt (**c**) (**GIFs 1-3**, resp.). Scale bar = 10 µm.

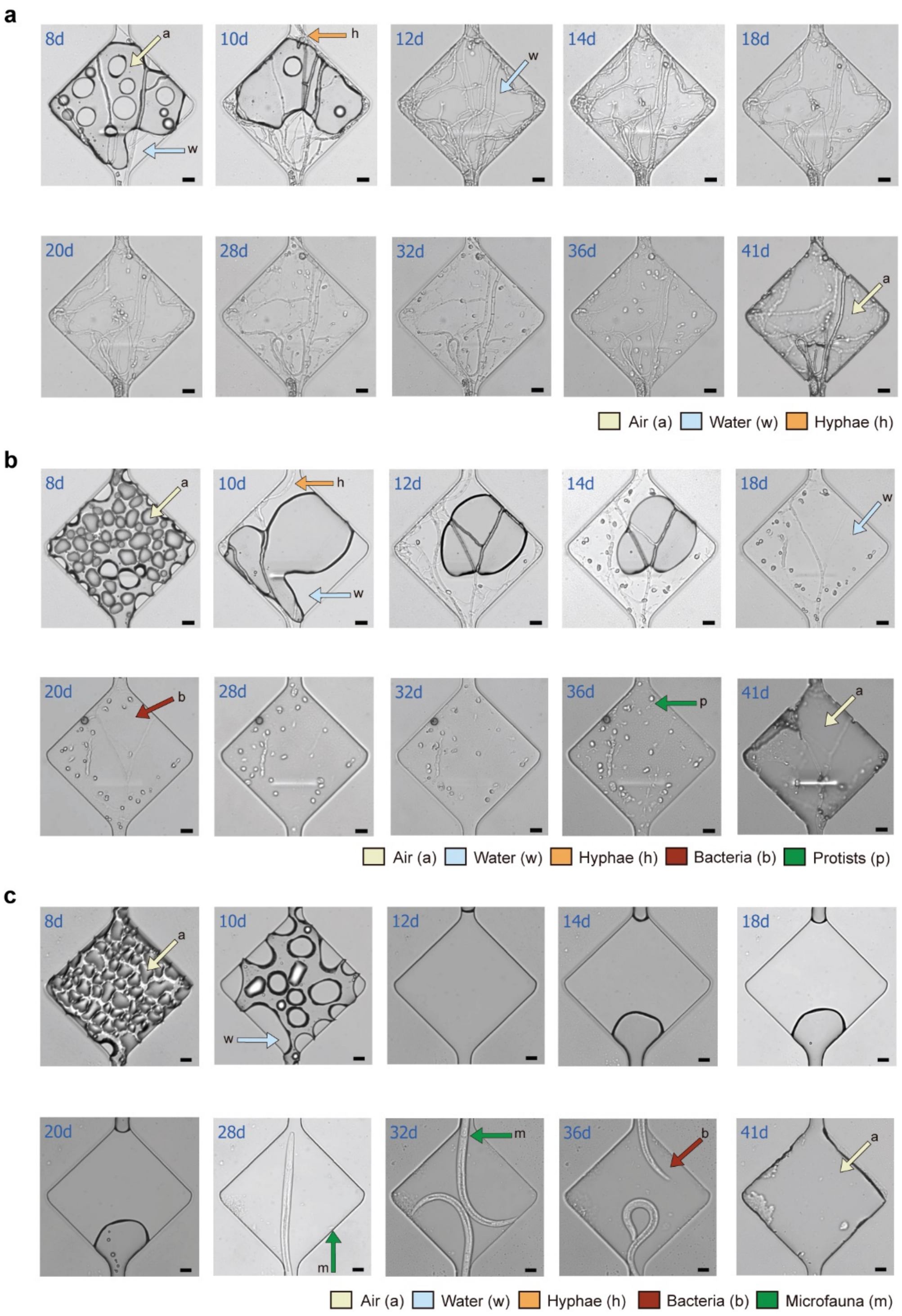

Supplementary Figure 4. Microbial succession in air-filled soil chips incubated with soil in the laboratory (Expt. 3). Image sequences of the time-resolved changes in microbial colonization of a diamond-shaped widening along the channels used to calculate the presence and abundance of major soil microbial groups and water films (**a**, **b**, **c**, corresponding to **GIFs 4-6**). Scale bar = 10 µm.

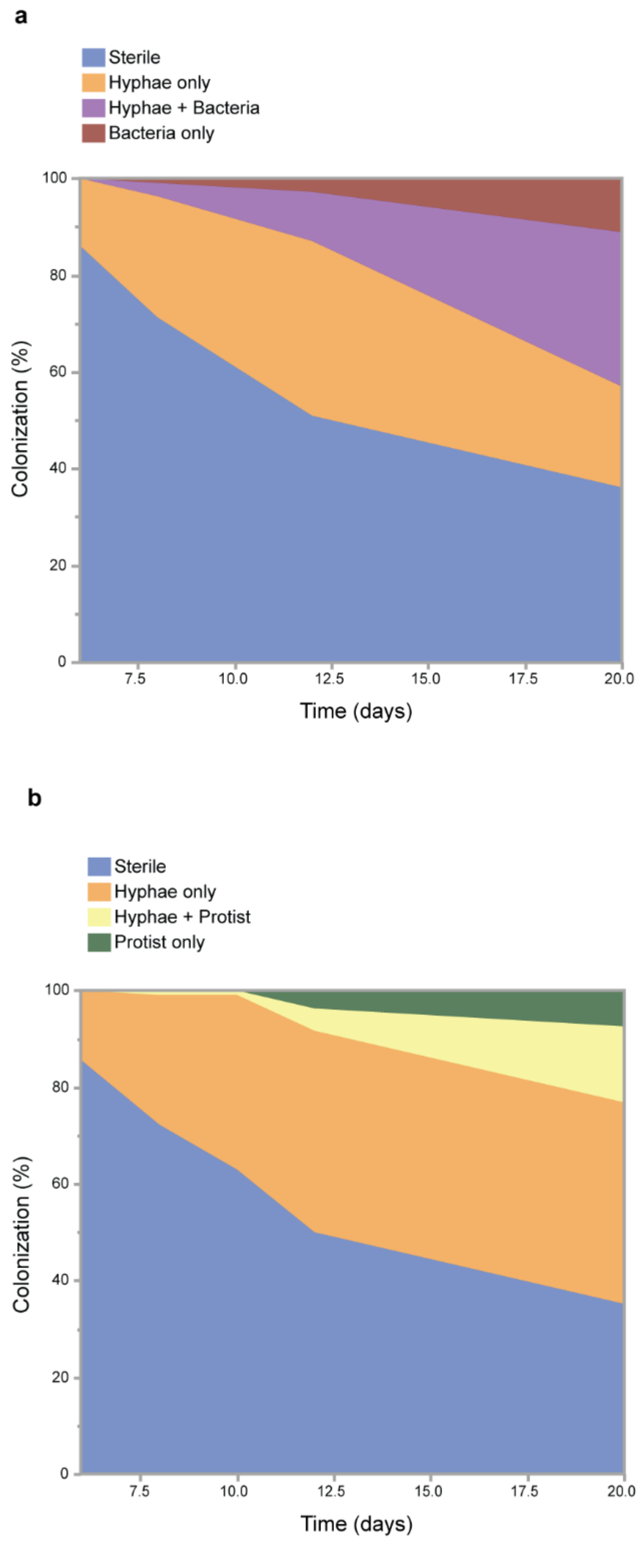

Supplementary Figure 5. Frequency of co-dispersal of bacteria and protists with fungal hyphae. Percentage of the 108 examined channels of three initially air-filled chips without any microorganism (sterile), with hyphae only, with bacteria or both (**a**), and with protists or both (**b**).
