# Supplementary material for: Microfluidic chips provide visual access to in situ soil ecology
Source: Commun Biol. 2021 Jul 20;4:889. doi: 10.1038/s42003-021-02379-5 (PMC8292388; doi:10.1038/s42003-021-02379-5)
Supplement: Supplementary file 3 — Description of Supplementary Files [file 42003_2021_2379_MOESM3_ESM.pdf]

Description of Additional Supplementary Files

File Name: Supplementary Data 1
Description: Data presented in Figures 2-5.

Supplementary Movies

1. **Supplementary Movie 1**. Water meniscus connecting soil particles and chip structures. The movie was recorded in an initially air-filled chip from experiment 1.
2. **Supplementary Movie 2**. Mobile soil particles blocking connectivity of channels. The movie was recorded in an initially malt medium-filled chip from experiment 1.
3. **Supplementary Movie 3**. Passage opening by hyphae that broke through the borders of an artificial pore space. The movie was recorded in an initially air-filled chip from experiment 1.
4. **Supplementary Movie 4**. Habitat fragmentation by hyphae. The movie was recorded in an initially air-filled chip from experiment 1.
5. **Supplementary Movie 5**. Hypha-facilitated bacterial dispersal across air gaps ("fungal highways"). The movie was recorded in an initially malt medium-filled chip from experiment
6. **Supplementary Movie 6**. Passage obstruction by a hypha that blocked all entrances of a rectangle pore. The movie was recorded in an initially malt medium-filled chip from experiment 1.
7. **Supplementary Movie 7**. Particle transportation by an amoeba. The movie was recorded in an initially water-filled chip from experiment 1.
8. **Supplementary Movie 8**. Flagellated protozoa foraging around soil aggregates. The movie was recorded in an initially malt medium-filled chip from experiment 1.
9. **Supplementary Movie 9**. Patchy bacterial colonization of water droplets. The movie was recorded in an initially air-filled chip from experiment 3.
10. **Supplementary Movie 10.** Microbial succession in the soil chips incubated with soil in the laboratory, air filled (Expt. 2). Image sequences of the time-resolved changes in microbial colonization of a diamond-shaped widening.
11. **Supplementary Movie 11.** Microbial succession in the soil chips incubated with soil in the laboratory, water filled (Expt. 2). Image sequences of the time-resolved changes in microbial colonization of a diamond-shaped widening.
12. **Supplementary Movie 12.** Microbial succession in the soil chips incubated with soil in the laboratory, malt medium filled (Expt. 2). Image sequences of the time-resolved changes in microbial colonization of a diamond-shaped widening.
13. **Supplementary Movie 13.** Microbial succession in air-filled soil chips incubated with soil in the laboratory (Expt. 3). Image sequences of the time-resolved changes in microbial colonization of a diamond-shaped widening.

14. **Supplementary Movie 14.** Microbial succession in air-filled soil chips incubated with soil in the laboratory (Expt. 3). Image sequences of the time-resolved changes in microbial colonization of a diamond-shaped widening.
15. **Supplementary Movie 15.** Microbial succession in air-filled soil chips incubated with soil in the laboratory (Expt. 3). Image sequences of the time-resolved changes in microbial colonization of a diamond-shaped widening.
16. **Supplementary Movie 16**. Displacement of bacteria and soil particles along a water flow. The movie was recorded in an initially air-filled chip from experiment 1.
17. **Supplementary Movie 17**. Particle tracking analysis within water stream channels. The movie was recorded in an initially air-filled chip from experiment 1.
18. **Supplementary Movie 18**. Particle tracking original movie, corresponding to Supplementary Movie 10. The movie was recorded in an initially air-filled chip from experiment 1.
19. **Supplementary Movie 19**. Obstruction of soil organisms such as nematodes and protists by hyphae. The movie was recorded in an initially air-filled chip from experiment 3.
20. **Supplementary Movie 20**. Ciliate pushing into soil particles and aggregates. The movie was recorded in an initially malt medium-filled chip from experiment 1.
21. **Supplementary Movie 21**. Bacterial accumulation around a fragmentated hypha. The movie was recorded in an initially malt medium-filled chip from experiment 1.
22. **Supplementary Movie 22**. Bacterial dispersal along a hypha in a channel is blocked by an air barrier. The movie was recorded in an initially air-filled chip from experiment 3.
