# Supplementary material for: Microfluidic chips provide visual access to in situ soil ecology
Source: Commun Biol. 2021 Jul 20;4:889. doi: 10.1038/s42003-021-02379-5 (PMC8292388; doi:10.1038/s42003-021-02379-5)
Supplement: Supplementary file 27 — Reporting Summary [file 42003_2021_2379_MOESM27_ESM.pdf]

nature research

Corresponding author(s): Edith Hammer
Paola Micaela Mafla Endara

Last updated by author(s): Jun 9, 2021

Reporting Summary

Nature Research wishes to improve the reproducibility of the work that we publish. This form provides structure for consistency and transparency in reporting. For further information on Nature Research policies, see our Editorial Policies and the Editorial Policy Checklist.

Statistics

For all statistical analyses, confirm that the following items are present in the figure legend, table legend, main text, or Methods section.

n/a	Confirmed	
☐	☒	The exact sample size (n) for each experimental group/condition, given as a discrete number and unit of measurement
☐	☒	A statement on whether measurements were taken from distinct samples or whether the same sample was measured repeatedly
☐	☒	The statistical test(s) used AND whether they are one- or two-sided<br>*Only common tests should be described solely by name; describe more complex techniques in the Methods section.*
☐	☒	A description of all covariates tested
☐	☒	A description of any assumptions or corrections, such as tests of normality and adjustment for multiple comparisons
☐	☒	A full description of the statistical parameters including central tendency (e.g. means) or other basic estimates (e.g. regression coefficient) AND variation (e.g. standard deviation) or associated estimates of uncertainty (e.g. confidence intervals)
☐	☒	For null hypothesis testing, the test statistic (e.g. F, t, r) with confidence intervals, effect sizes, degrees of freedom and P value noted<br>*Give P values as exact values whenever suitable.*
☒	☐	For Bayesian analysis, information on the choice of priors and Markov chain Monte Carlo settings
☒	☐	For hierarchical and complex designs, identification of the appropriate level for tests and full reporting of outcomes
☒	☐	Estimates of effect sizes (e.g. Cohen's d, Pearson's r), indicating how they were calculated

Our web collection on statistics for biologists contains articles on many of the points above.

Software and code

Policy information about availability of computer code

Data collection	Chip design was performed in AutoCAD 2015. The commercial software NIS Elements advanced research imaging software (Nikon) was used for microscope image acquisition.
Data analysis	Particle tracking image analysis was performed with Fiji Image J 1.52i, plug-in TrackMate version 3.8.0, statistical analysis was performed in JMP pro 15.0 (SAS Institute Inc., Cary, USA), figures were prepared with help of the Adobe Suite.

For manuscripts utilizing custom algorithms or software that are central to the research but not yet described in published literature, software must be made available to editors and reviewers. We strongly encourage code deposition in a community repository (e.g. GitHub). See the Nature Research guidelines for submitting code & software for further information.

Data

Policy information about availability of data

All manuscripts must include a data availability statement. This statement should provide the following information, where applicable:

- Accession codes, unique identifiers, or web links for publicly available datasets
- A list of figures that have associated raw data
- A description of any restrictions on data availability

The datasets generated during and/or analysed during the current study presented in Figures 2-5 are included as a supplementary file. Image/video documentations and other datasets are available from the corresponding author upon reasonable request.

Field-specific reporting

Please select the one below that is the best fit for your research. If you are not sure, read the appropriate sections before making your selection.

☐ Life sciences ☐ Behavioural & social sciences ☒ Ecological, evolutionary & environmental sciences

For a reference copy of the document with all sections, see nature.com/documents/nr-reporting-summary-flat.pdf

Ecological, evolutionary & environmental sciences study design

All studies must disclose on these points even when the disclosure is negative.

Study description	We study soil microbial dispersal and their interactions with each other and the simulated soil pore space of microfluidic chips. Expt 1 is performed in situ with chips buried in the soil for two months, Expts 2 & 3 use soil inoculum for time-resolved studies in the laboratory.
Research sample	Soil including its microorganisms
Sampling strategy	Chips were buried for Expt. 1 in a small groove of deciduous trees in the city of Lund, and soil samples were taken for Expts 2-3 from an adjacent meadow, Sweden, 55° 42´ 49.5´´ N, 13° 12´ 32.5´´ E.
Data collection	Data on microbial dispersal and interactions was recorded by counting organisms at distinct spatial locations marked in the chips under the microscope by the same person, and documented via images and videos.
Timing and spatial scale	Chips buried in the soil (horizontally at 10 cm depth) were recovered and examined after 2 months; soil-inoculated chips in the laboratory were monitored over 36 days at 6, 8, 10, 12, 20, 28 and 36 days.
Data exclusions	One replicate of water-filled chips in Expt. 2, and one measurement data point (1 replicate at day 20) in Expt.3 had to be excluded due to treatment failure.
Reproducibility	The field experiment was prepared via two pilot studies that showed similar results, and the laboratory experiments reproduced the findings of the field study, thus corroborating each other.
Randomization	Chips were incubated at random locations in the groove of deciduous trees in treatment groups (three pore space filling levels); internal replication of the channel types was randomized via a script provided by urbanlisp (http://www.urbanlisp.com).
Blinding	Treatment types were blinded during data acquisition.

Did the study involve field work? ☒ Yes ☐ No

Field work, collection and transport

Field conditions	Chip incubation took place October-December 2017, and did not involve soil freezing.
Location	Chips were incubated in the soil of a small groove of deciduous trees in the city of Lund, Sweden, 55° 42´ 49.5´´ N, 13° 12´ 32.5´´ E
Access & import/export	The field site is part of the biology campus, Lund University, and samples needed only be transported to the lab by walking
Disturbance	Small holes were dug into the soil (ca. 20 cm diameter, 10 cm depth) and carefully restored.

Reporting for specific materials, systems and methods

We require information from authors about some types of materials, experimental systems and methods used in many studies. Here, indicate whether each material, system or method listed is relevant to your study. If you are not sure if a list item applies to your research, read the appropriate section before selecting a response.

Materials & experimental systems

n/a	Involved in the study	
☒	☐	Antibodies
☒	☐	Eukaryotic cell lines
☒	☐	Palaeontology and archaeology
☐	☒	Animals and other organisms
☒	☐	Human research participants
☒	☐	Clinical data
☒	☐	Dual use research of concern

Methods

n/a	Involved in the study	
☒	☐	ChIP-seq
☒	☐	Flow cytometry
☒	☐	MRI-based neuroimaging

Animals and other organisms

Policy information about studies involving animals; ARRIVE guidelines recommended for reporting animal research

Laboratory animals	The study did not involve laboratory animals
Wild animals	The study did not involve wild animals
Field-collected samples	Soil including its natural microbial community was used as inoculum for the microfluidic chips
Ethics oversight	No ethical approval or guidance was required because there are no ethical implications associated with the microbial organisms

Note that full information on the approval of the study protocol must also be provided in the manuscript.
